# Supplementary material for: Conserved anti-inflammatory effects and sensing of butyrate in zebrafish
Source: Gut Microbes. 2020 Oct 16;12(1):1824563. doi: 10.1080/19490976.2020.1824563 (PMC7575005; doi:10.1080/19490976.2020.1824563)
Supplement: Supplemental Material [file KGMI_A_1824563_SM3703.zip › Supplementary information/Zebrafish hcar1 supplement.docx]

**Zebrafish hcar1a genomic sequence**

Hcar1a

>10 dna:chromosome chromosome:GRCz11:10:44449582:44450514:1

ATGAACAACTCCTCATTGTGCTGCACCTATGAAACCCCCCTGCTGGATGCATATCTGCCCCCTGTTCTGTTCAGCGAGTTCGTCCTGGGCCTGATGGGGAACGGTCTGGCCCTCTGCATGTTCTTCTTCCACAGGGACTCCTGGAAGCCCAACTCCATCTATCTGGCTCATTTGGCTCTGGCCAACTCGCTGGTGCTGTTCTGCCTTCCGTTCCGGGCTGACTACTACCGGCGGGGCAAGCACTGGGTCTACGGGGACGCTTTCTGCCGTGTGCTGCTGTTTCTGCTGGCCGCCAATCGAGCCGCCGGTATCTTCTTCCTGACGGCGGTGGCTGTGGACCGATACCTGAAGATCGTGCACCCGCTGAACCGCATCAACCAAATGGGTCTGCGCTACGCCCTGTGGGTGTCTGTGGGCCTGTGGGCGCTCATCATAGCCATGACCGTATATCTGCTCGCCGATAAACACTTCTACTATCTGAACAACCACACTCAATGCGAAAGCTTCAATATTTGCTTTGGGAGCAACTCTCGCTTCACTTGGCATAATGTGTTTTACGTTATTCAATTCTTCGTGCCGACGTTCATCGTCATCTACTGCTCCACATGTATAACCTGGCAGCTGAAGGGTAAAACAATTGACAAGCACGGTAAAATAAGAAGAGCTGTGCGGTTTGTGTTAGCAGTGGCTTTGGTTTTTATCATATGCTTCTTCCCGAGTAACATCTCTCGAATCTCGATGTATGTTCTTAAACTCTGGTATAACGAATGCCAGTATTTTAGTGACGCAAATGATGCATTTAAAACCACAGTCTGCTTTACGTACTTCAACAGCGTGCTCAATCCTGTGGTCTACTACTTCTCCAGCCCTGCCGTCAGCGGATCCTTGAGGAAAATATATATGAGGATTTTAGGACAGAAGATTGAGGAGTGA

**Zebrafish hcar1b genomic sequence**

>10 dna:chromosome chromosome:GRCz11:10:44457484:44458418:1

ATGAACAACTCCTCCTCGGTGTGCTGCGCTTTCGACGCTCCCATTCTGGATGAGGTGTTACCCCCTGTTCTGTTCAGCGAGTTCGTCCTGGGCCTGATGGGGAACGGTCTGGCCCTCTGCATGTTCTTCTTCCACAGGGACTCCTGGAAGCCCAACTCCATCTATCTGGCTCATCTGGCTCTGGCCGACTCGCTGGTGCTGTTCTGCCTTCCGTTCCGGGCTGACTACTACCGGCGGGGCAAGCACTGGGTCTACGGGGACGCTTTCTGCCGTGTGCTGCTGTTTCTGCTGGCTGCAAATCGAGCCGCCGGTATCTTCTTCCTGACGGCGGTGGCTGTGGACCGATACCTGAAGATCGTGCACCCGCTGAACCCCATCAACCAAATGGGTCTGCGCTACGCCCTGTGGGTGTCTGTGGGCCTGTGGGCGCTCATCATAGCCATGACCGTATATCTGCTCGCCGATAAACACTTCTACTATCGAAATAACCGAACGCAGTGCGAAAGCTTCAATATCTGTTTAGGACACAACGCTCTGTCTGATTGGCACAATTCGTTTTACGTTATTCAATTCTTCGTGCCGACGTTCATCGTCATCTACTGCTCCACATGTATAACCTGGCAGCTGAAGGGTAAAACAATTGACAAGCACGGTAAAATAAGAAGAGCTGTGCGGTTTGTGTTAGCAGTGGCTTTGGTTTTCATCATATGCTTCTTCCCCAGTAATGTCTCCCGTATTGCTGTTTGGGTTCTGAAAAGCTGGCATAATGAATGTCAATATTTTCAGGATGCAAATGTGGCGTTTTATATCACCGTCTGCTTTACGTACTTCAACAGCGTGCTCAATCCTGTGGTCTACTACTTCTCCAGCCCTGCCGTCAGCAGATCCTTGAGGAAAATATATATGAGGATGTCAGGACAGAAGATTGAGGATGA

gRNA target

qFw primer binding site

qRv primer binding site

**hcar1a gRNA mismatches to hcar1b sequence**

Target 1

A – **C**AATCGAGCCGCCGGTA

B – **A**AATCGAGCCGCCGGTA

Target 2 – no clear homology at site

Target 3

A – AGTAA**CA**TCTC**T**CG**A**AT**C**

B – AGTAA**TG**TCTC**C**CG**T**AT**T**

**Alignment of hcar1a (top) to hcar1b (bottom)**

Matches:864; Mismatches:62; Gaps:16; Unattempted:0

* * * * * * * * * *

933<TCACTCCTCAATCTTCTGTCCTAAAATCCTCATATATATTTTCCTCAAGGATCCGCTGACGGCAGGGCTGGAGAAGTAGTAGACCACAGGATTGAGCACG<834

935<TCA-TCCTCAATCTTCTGTCCTGACATCCTCATATATATTTTCCTCAAGGATCTGCTGACGGCAGGGCTGGAGAAGTAGTAGACCACAGGATTGAGCACG<837

* * * * * * * * * *

833<CTGTTGAAGTACGTAAAGCAGACTGTGGTTTTAAATGCATCATTTGCGTCACT-AAAATACTGGCATTCGTTATACCAGAGTTTAAGAA--CATACATCG<737

836<CTGTTGAAGTACGTAAAGCAGACGGTGATATAAAACGCCACATTTGCATC-CTGAAAATATTGACATTCATTATGCCAGCTTTTCAGAACCCAAACAGC-<739

* * * * * * * * * *

736<AGATTCGAGAGATGTTACTCGGGAAGAAGCATATGATAAAAACCAAAGCCACTGCTAACACAAACCGCACAGCTCTTCTTATTTTACCGTGCTTGTCAAT<637

738<A-ATACGGGAGACATTACTGGGGAAGAAGCATATGATGAAAACCAAAGCCACTGCTAACACAAACCGCACAGCTCTTCTTATTTTACCGTGCTTGTCAAT<640

* * * * * * * * * *

636<TGTTTTACCCTTCAGCTGCCAGGTTATACATGTGGAGCAGTAGATGACGATGAACGTCGGCACGAAGAATTGAATAACGTAAAACACATTATGCCAAGTG<537

639<TGTTTTACCCTTCAGCTGCCAGGTTATACATGTGGAGCAGTAGATGACGATGAACGTCGGCACGAAGAATTGAATAACGTAAAACGAATTGTGCCAA-TC<541

* * * * * * * * * *

536<A-AGCGAGAGTTG-CTCCCAAAGCAAATATTGAAGCTTTCGCATTGAGTGTGGTTGTTCAGATAGTAGAAGTGTTTATCGGCGAGCAGATATACGGTCAT<439

540<AGACAGAGCGTTGTGTCCTAAA-CAGATATTGAAGCTTTCGCACTGCGTTCGGTTATTTCGATAGTAGAAGTGTTTATCGGCGAGCAGATATACGGTCAT<442

* * * * * * * * * *

438<GGCTATGATGAGCGCCCACAGGCCCACAGACACCCACAGGGCGTAGCGCAGACCCATTTGGTTGATGCGGTTCAGCGGGTGCACGATCTTCAGGTATCGG<339

441<GGCTATGATGAGCGCCCACAGGCCCACAGACACCCACAGGGCGTAGCGCAGACCCATTTGGTTGATGGGGTTCAGCGGGTGCACGATCTTCAGGTATCGG<342

* * * * * * * * * *

338<TCCACAGCCACCGCCGTCAGGAAGAAGATACCGGCGGCTCGATTGGCGGCCAGCAGAAACAGCAGCACACGGCAGAAAGCGTCCCCGTAGACCCAGTGCT<239

341<TCCACAGCCACCGCCGTCAGGAAGAAGATACCGGCGGCTCGATTTGCAGCCAGCAGAAACAGCAGCACACGGCAGAAAGCGTCCCCGTAGACCCAGTGCT<242

* * * * * * * * * *

238<TGCCCCGCCGGTAGTAGTCAGCCCGGAACGGAAGGCAGAACAGCACCAGCGAGTTGGCCAGAGCCAAATGAGCCAGATAGATGGAGTTGGGCTTCCAGGA<139

241<TGCCCCGCCGGTAGTAGTCAGCCCGGAACGGAAGGCAGAACAGCACCAGCGAGTCGGCCAGAGCCAGATGAGCCAGATAGATGGAGTTGGGCTTCCAGGA<142

* * * * * * * * * *

138<GTCCCTGTGGAAGAAGAACATGCAGAGGGCCAGACCGTTCCCCATCAGGCCCAGGACGAACTCGCTGAACAGAACAGGGGGCAGATA-TGCATCCAGCAG<40

141<GTCCCTGTGGAAGAAGAACATGCAGAGGGCCAGACCGTTCCCCATCAGGCCCAGGACGAACTCGCTGAACAGAACAGGGGGTA-ACACCTCATCCAGAAT<43

* * *

39<GGGGGTTTCATAGGTGCAGCACA---ATGAGGAGTTGTTCAT<1

42<GGGAGCGTCGAAAGCGCAGCACACCGAGGAGGAGTTGTTCAT<1

**Predicted Hcar1a**

MNNSSLCCTYETPLLDAYLPPVLFSEFVLGLMGNGLALCMFFFHRDSWKPNSIYLAHLALANSLVLFCLPFRADYYRRGKHWVYGDAFCRVLLFLLAANRAAGIFFLTAVAVDRYLKIVHPLNRINQMGLRYALWVSVGLWALIIAMTVYLLADKHFYYLNNHTQCESFNICFGSNSRFTWHNVFYVIQFFVPTFIVIYCSTCITWQLKGKTIDKHGKIRRAVRFVLAVALVFIICFFPSNISRISMYVLKLWYNECQYFSDANDAFKTTVCFTYFNSVLNPVVYYFSSPAVSGSLRKIYMRILGQKIEE*

**Predicted Hcar1b**

MNNSSSVCCAFDAPILDEVLPPVLFSEFVLGLMGNGLALCMFFFHRDSWKPNSIYLAHLALADSLVLFCLPFRADYYRRGKHWVYGDAFCRVLLFLLAANRAAGIFFLTAVAVDRYLKIVHPLNPINQMGLRYALWVSVGLWALIIAMTVYLLADKHFYYRNNRTQCESFNICLGHNALSDWHNSFYVIQFFVPTFIVIYCSTCITWQLKGKTIDKHGKIRRAVRFVLAVALVFIICFFPSNVSRIAVWVLKSWHNECQYFQDANVAFYITVCFTYFNSVLNPVVYYFSSPAVSRSLRKIYMRMSGQKIED

**Alignment Hcar1a and Hcar1b amino acid sequence**

Identities:275/309(89%), Positives:289/309(93%), Gaps:0/309(0%)

Hcar1a 2 NNSSLCCTYETPLLDAYLPPVLFSEFVLGLMGNGLALCMFFFHRDSWKPNSIYLAHLALA 61

N+SS+CC ++ P+LD LPPVLFSEFVLGLMGNGLALCMFFFHRDSWKPNSIYLAHLALA

Hcar1b 3 NSSSVCCAFDAPILDEVLPPVLFSEFVLGLMGNGLALCMFFFHRDSWKPNSIYLAHLALA 62

Hcar1a 62 NSLVLFCLPFRADYYRRGKHWVYGDAFCRVLLFLLAANRAAGIFFLTAVAVDRYLKIVHP 121

+SLVLFCLPFRADYYRRGKHWVYGDAFCRVLLFLLAANRAAGIFFLTAVAVDRYLKIVHP

Hcar1b 63 DSLVLFCLPFRADYYRRGKHWVYGDAFCRVLLFLLAANRAAGIFFLTAVAVDRYLKIVHP 122

Hcar1a 122 LNRINQMGLRYALWVSVGLWALIIAMTVYLLADKHFYYLNNHTQCESFNICFGSNSRFTW 181

LN INQMGLRYALWVSVGLWALIIAMTVYLLADKHFYY NN TQCESFNIC G N+ W

Hcar1b 123 LNPINQMGLRYALWVSVGLWALIIAMTVYLLADKHFYYRNNRTQCESFNICLGHNALSDW 182

Hcar1a 182 HNVFYVIQFFVPTFIVIYCSTCITWQLKGKTIDKHGKIRRAVRFVLAVALVFIICFFPSN 241

HN FYVIQFFVPTFIVIYCSTCITWQLKGKTIDKHGKIRRAVRFVLAVALVFIICFFPSN

Hcar1b 183 HNSFYVIQFFVPTFIVIYCSTCITWQLKGKTIDKHGKIRRAVRFVLAVALVFIICFFPSN 242

Hcar1a 242 ISRISMYVLKLWYNECQYFSDANDAFKTTVCFTYFNSVLNPVVYYFSSPAVSGSLRKIYM 301

+SRI+++VLK W+NECQYF DAN AF TVCFTYFNSVLNPVVYYFSSPAVS SLRKIYM

Hcar1b 243 VSRIAVWVLKSWHNECQYFQDANVAFYITVCFTYFNSVLNPVVYYFSSPAVSRSLRKIYM 302

Hcar1a 302 RILGQKIEE 310

R+ GQKIE+

Hcar1b 303 RMSGQKIED 311
