# Supplementary figures and images for: Conserved anti-inflammatory effects and sensing of butyrate in zebrafish
Source: Gut Microbes. 2020 Oct 16;12(1):1824563. doi: 10.1080/19490976.2020.1824563 (PMC7575005; doi:10.1080/19490976.2020.1824563)

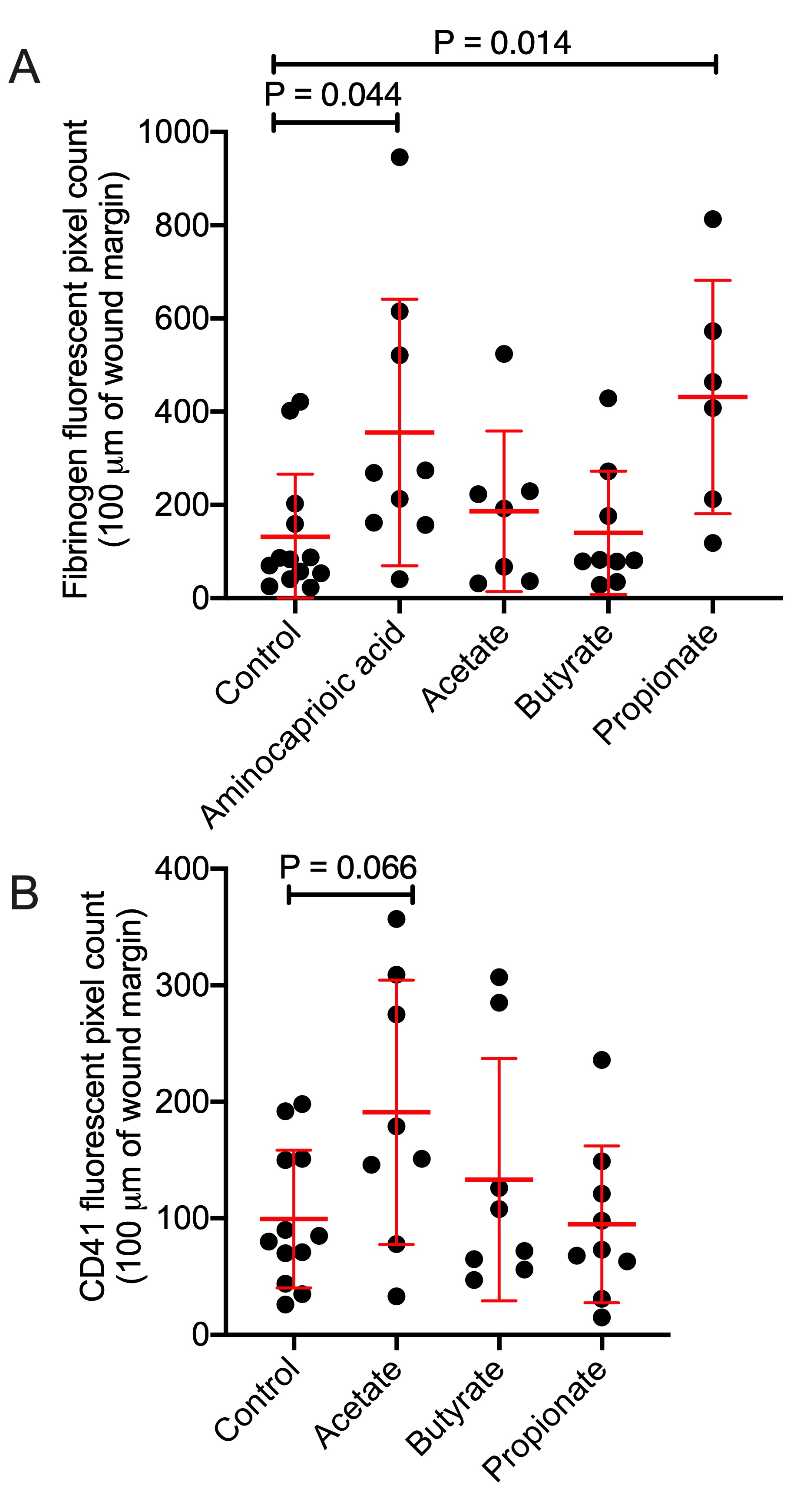

Supplement: Supplemental Material [file KGMI_A_1824563_SM3703.zip › Supplementary information/Supplementary Figure 1.jpg]

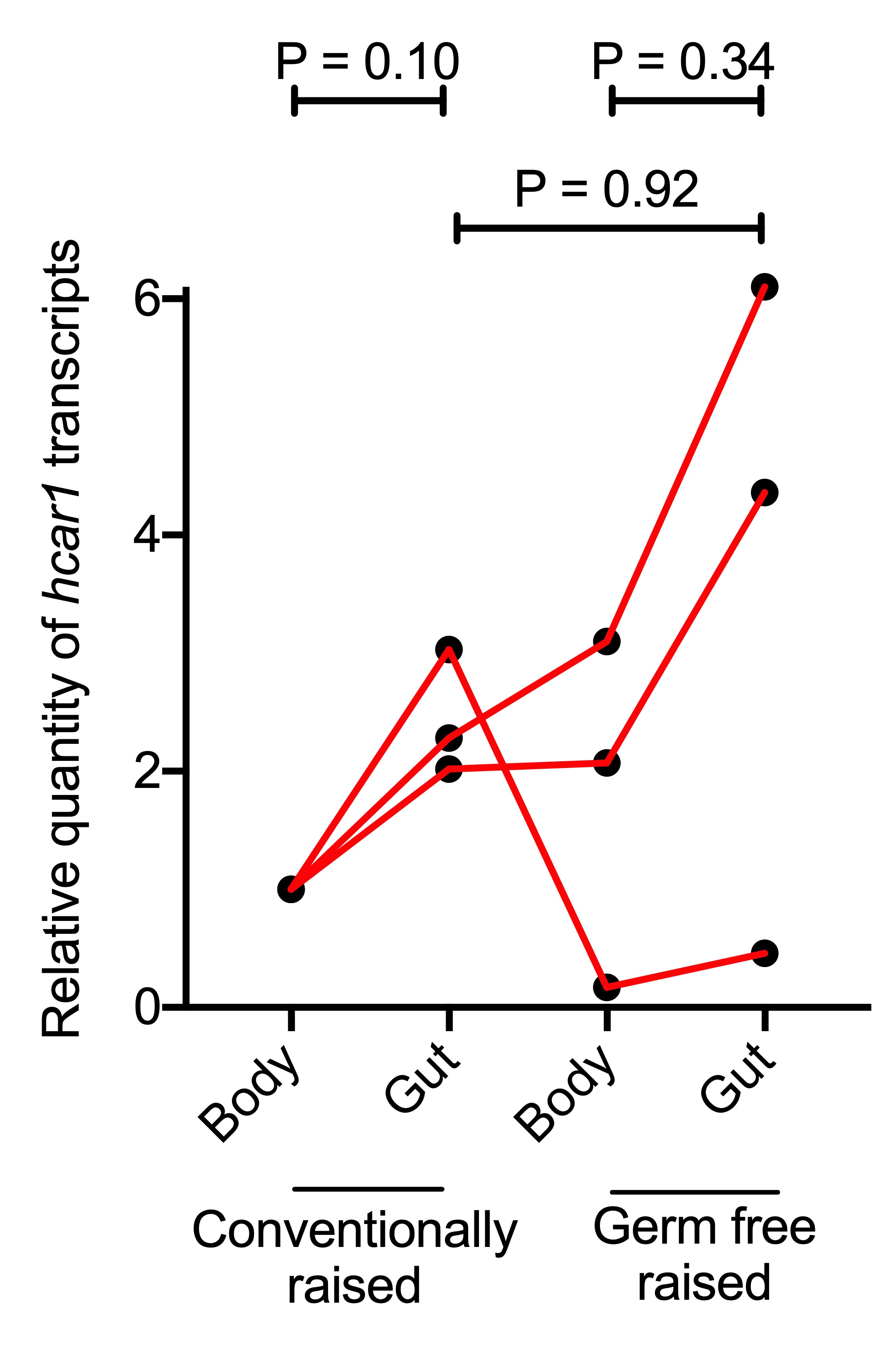

Supplement: Supplemental Material [file KGMI_A_1824563_SM3703.zip › Supplementary information/Supplementary Figure 2.jpg]

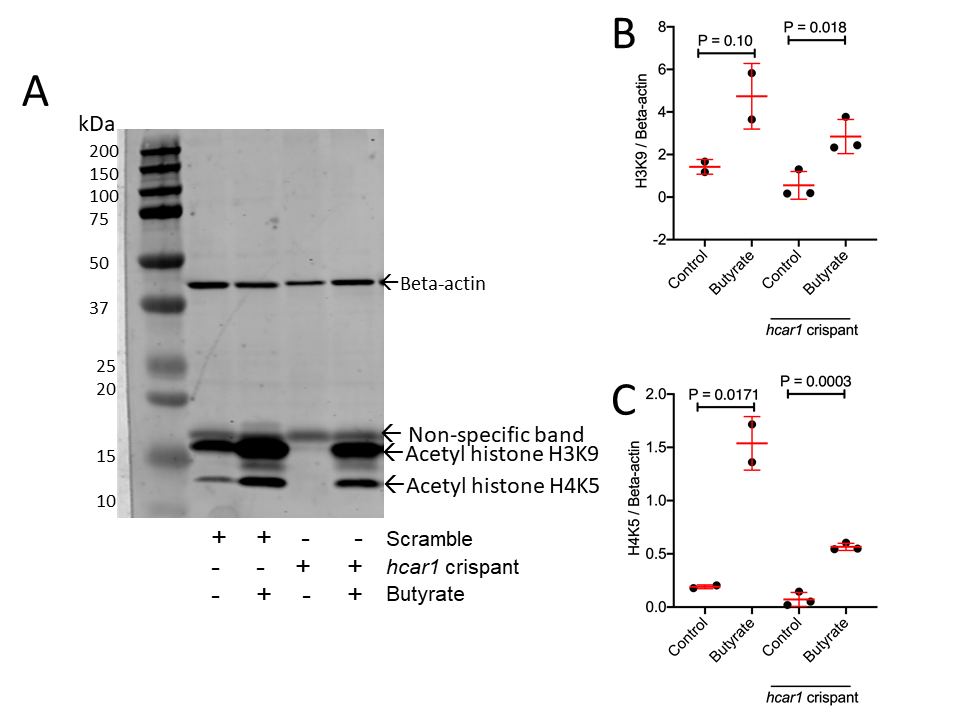

Supplement: Supplemental Material [file KGMI_A_1824563_SM3703.zip › Supplementary information/Supplementary Figure 3.tif]
